# Necrotizing Fasciitis of the Upper Extremity, Case Report and Review of the Literature

**DOI:** 10.5812/traumamon.6398

**Published:** 2012-07-31

**Authors:** Shahram Nazerani, Ahmad Maghari, Mohammad Hosein Kalantar Motamedi, Jalal Vahedian Ardakani, Nikdokht Rashidian, Tina Nazerani

**Affiliations:** 1 Department of Surgery, Tehran University of Medical Sciences, Firuzgar Medical Center, Tehran, IR Iran; 2 Department of Reconstructive Surgeries, Mehr General Hospital, Tehran, IR Iran; 3 Trauma Research Center, Baqiyatallah University of Medical sciences, Tehran, IR Iran

**Keywords:** Fasciitis, Necrotizing

## Abstract

Necrotizing fasciitis is a rare, life-threatening infection most commonly seen in patients with diabetes mellitus, intravenous drug abuse, and immunocompromised conditions. The extremities are the primary sites of involvement in as many as two thirds of the cases. In a significant proportion of patients, the extremities are involved as a result of trauma, needle puncture or extravasation of drugs. The infection is usually polymicrobial. Treatment involves broad-spectrum antibiotics and multiple surgical debridements or amputation. We present a patient with necrotizing fasciitis of the upper limb and present our experience with this often lethal condition.

## 1. Introduction

Necrotizing fasciitis is a rapidly progressive, potentially lethal bacterial infection involving skin, subcutaneous tissue, and superficial fascia. The incidence of this type of infection has been estimated to be around 4 per million of the population and it has increased over the last decade (1). Necrotizing fasciitis encompasses a broad spectrum of presentations, and is associated with a poor outcome (2). The elderly, diabetic, intravenous drug abusers, immunocompromised and other at risk groups are more susceptible to developing necrotizing fasciitis. It can affect any body part; however, commonly the extremities, abdominal wall and perineum are favored.

The pathophysiology of necrotizing fasciitis is complex and there is most often a polymicrobial basis (3). Depending on the causative organisms, necrotizing fasciitis is categorized as Type I, II or III. Type I is a mixed infection caused by aerobic and anaerobic bacteria. Type II is caused by anaerobic group A streptococci, possibly with co-infection by S. aureus. Type III of necrotizing infection is caused by the marine vibrios (Gram-negative rods). The entry portal for these bacteria is a puncture from fish or marine insects (4).

Necrotizing fasciitis may result from any injury to the skin or from hematogenous spread. Any major or minor trauma compromising skin integrity such as needle puncture, insect bites, burns, lacerations, extravasation of drugs, surgical wound, blunt trauma etc. may develop this insidiously advancing soft-tissue condition (5). The extremities are the primary sites of infection in as many as two thirds of the cases, with lower extremity being slightly more prevalent. Because necrotizing fasciitis is relatively rare, few studies have investigated upper extremity alone. A systematic review of the literature in order to investigate the special characteristics of necrotizing fasciitis affecting the upper and lower extremities, found a 22% mortality rate and a 22% rate of amputation of extremities with necrotizing fasciitis (6).

## 2. Case Report

The patient was a 66 year-old male who was admitted to our hospital with extensive cellulitis and fasciitis of his left chest wall and axillary region. He mentioned trauma to his left arm 2 months ago which had caused a swelling and tenderness in his left arm. In another hospital he had undergone several debridements of soft tissue necrosis and broad spectrum antibiotic therapy. The patient had been diagnosed having necrotizing fasciitis at the previous hospital and the “finger probe test” was positive which identifies the disease.

**Figure 1:**
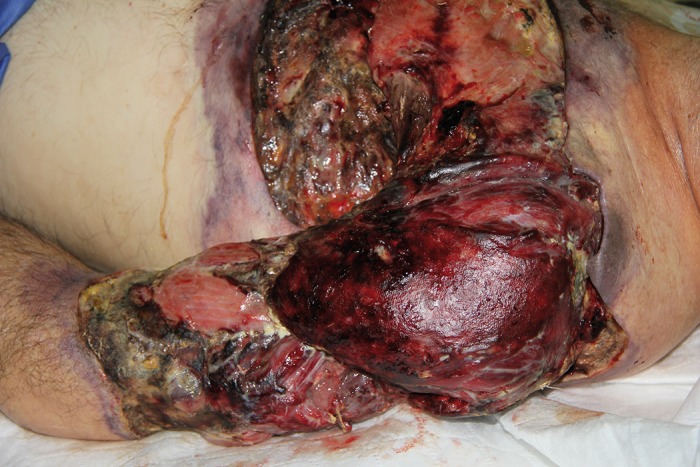
Necrotizing fasciitis of the upper part of the left arm.

**Figure 2:**
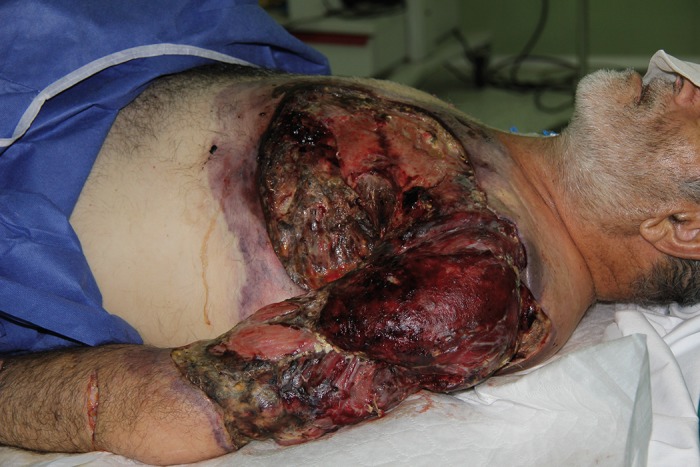
Extent of necrosis.

**Figure 3:**
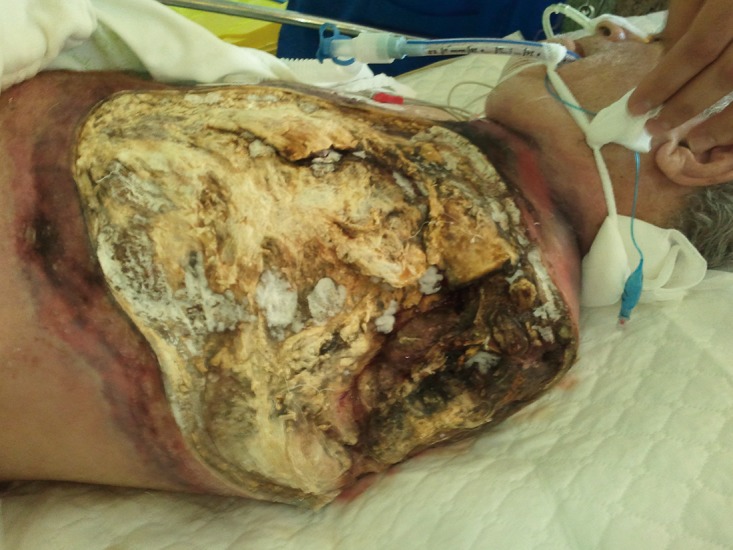
Progression of necrosys despite limb amputation.

He had a history of uncontrolled diabetes mellitus and coronary artery disease (Figure 1,2,3). The patient was transferred to the ICU and after evaluating the wound the decision to amputate the extremity was made and amputation was done the day after admission. In order to control his infection after taking multiple cultures from his wound, blood and urine, high-dose clindamycin, vancomycin and 24 million units of crystal penicillin was started. The patient was admitted on an emergency basis and before operation the lab tests were: Hb: 8 mg/dl, WBC 25000, BUN 10, creatinine 1.1 mg/dl; the cultures grew a mixed bacteria, consisting of methicillin resistant Staphylococcus aureus, Streptococci and Pseudomonas aureoginosa resistant to all the antibiotics. The blood and urine cultures were negative since the patient was receiving high doses of antibiotics. During the first postoperative day the patient developed a low hemoglobin and a 2.5 mg/dl creatinine, thus the antibiotic regimen was adjusted accordingly. The patient also had a post operative blood sugar of 331 mg/dl.

The patient became lethargic and because of his respiratory distress he was intubated and mechanical ventilation was started. The blood test revealed a white cell count of 40,000/dl and hemoglobin of 9 g/dl. His ABG also showed compensated metabolic acidosis. On the second postoperative day the patient’s metabolic acidosis progressed and because of increasing creatinine levels acute tubular necrosis was diagnosed. He received 2 units of packed cell and Lasix. On the 3rd postoperative day he developed a low blood pressure which was unresponsive to dopamine drip. On the same day the patient had bradycardia and asystole and underwent CPR. The CPR was unsuccessful and the patient passed away.

## 3. Discussion

Necrotizing fasciitis is a rare life-threatening soft tissue infection in which inflammation and pus rapidly spread along fascial planes (7). The incidence of necrotizing fasciitis has been estimated to lie between 0.4 and 0.53 cases per 100.000 of the population. Recently an increasing incidence has been reported, attributed to a better reporting system and an increase in strains associated with necrotizing fasciitis due to antibiotic abuse (4). Recognized predisposing factors include increasing age, disease or treatment causing immunosuppression. Trauma may precede necrotizing fasciitis. The site of entry of bacteria can be a raw area, a puncture, a laceration, or a surgical wound (8, 9).

Review of the literature demonstrates a wide variety of different presentations. As the process begins in the deep subcutaneous tissue and fascia, early clinical recognition may be difficult (10). The most common initial sign is an erythematous area of cellulitis with severe tenderness. Other common signs are edema, induration of the skin, purulence, fluctuation, and local warmth as well. These signs are easily confused with other, less serious conditions such as cellulitis or abscess. A patient complaining of severe pain out of proportion to the apparent severity of the lesion should alert the physician to possible diagnosis of necrotizing fasciitis, as early diagnosis and management is vital (6). In addition to disproportionate pain, patches of necrosis due to thrombosis of nutrient vessels to the skin, hypotension, and the clinical sign of gas formation are clues that help to establish the diagnosis of necrotizing fasciitis (11). Tissue crepitus is highly suggestive of necrotizing fasciitis and represents gas formation in the soft tissue. The classic “hard signs” of skin necrosis and crepitus are found in fewer than 1 in 10 patients. Systemic evidence of sepsis, such as hypotension, tachycardia, and hyperthermia are alarming signs (12).

Radiological investigations can also be used to help diagnosis. Plain X-ray may detect soft tissue gas even when there is no palpable crepitus. Computed tomography has been used to investigate the extent of disease, but magnetic resonance imaging has been proven to be highly sensitive in the diagnosis of necrotizing fasciitis (13). As a more direct diagnostic procedure some authors have advocated the “finger probe test”. This involves a 2 cm vertical incision through the skin down to the deep fascia. A finger is then introduced into the wound and blunt dissection is performed. In healthy tissue the subcutaneous tissue adhere tightly to deep fascial layers whereas in necrotizing fasciitis blunt dissection will easily separate layers (1).

When the diagnosis of necrotizing fasciitis is established, urgent exploration and debridement is imperative. Intravenous antibiotic should be administrated empirically as soon as possible. This medication should have a broad antimicrobial spectrum, and should cover likely pathogens (14). Intravenous immunoglobulin (Ig) has been used as well in the treatment of necrotizing fasciitis and sporadic reports have demonstrated reduced mortality in patients receiving IV Ig (4). In most cases repeated debridement is mandatory. All necrotic tissue must be excised and surgical incisions should be generous in order to extend beyond the necrotic area (15). Amputation of a limb may be unavoidable when there is no viable tissue left or in the event of an uncontrolled infection that predisposes to toxic shock. Regardless of treatments, necrotizing fasciitis has an overall mortality of 22 % of patients with necrotizing fasciitis of extremities (6).

## 4. Conclusions

Upper limb necrotizing fasciitis is a rare presentation of this life-threatening disease. An early diagnosis and subsequent prompt treatment is essential because of the high mortality rate of the disease. As the initial presentations of the patients may be confusing, the early use of laboratory and radiological investigations to confirm and establish an early diagnosis is recommended. Once the diagnosis is made early use of IV broad-spectrum antibiotics and surgical treatment is crucial, however still there is a chance of high morbidity and mortality.
